# Traditional Chinese medicine Lingguizhugan decoction ameliorate HFD-induced hepatic-lipid deposition in mice by inhibiting STING-mediated inflammation in macrophages

**DOI:** 10.1186/s13020-021-00559-3

**Published:** 2022-01-05

**Authors:** Lin Cao, Erjin Xu, Rendong Zheng, Zhili Zhangchen, Rongling Zhong, Fei Huang, Juan Ye, Hongping Sun, Yaofu Fan, Shaofeng Xie, Yu Chen, Yijiao Xu, Jing Cao, Wen Cao, Chao Liu

**Affiliations:** 1 Affiliated Hospital of Integrated Traditional Chinese and Western Medicine, Nanjing University of Chinese Medicine, No. 100, Shizi Street, Hongshan Road, Nanjing, 210028 China; 2 Jiangsu Province Academy of Traditional Chinese Medicine, No. 100, Shizi Street, Hongshan Road, Nanjing, 210028 China; 3 Suzhou Traditional Chinese Medicine Hospital Affiliated to Nanjing University of Chinese Medicine, No. 18 Yangsu Road, Gusu District, Suzhou, 215002 China; 4 Affiliated Hospital of Nanjing University of Chinese Medicine, 155 Hanzhong Road, Qinhuai District, Nanjing, 210029 China; 5 The Affiliated Jiangning Hospital of Nanjing Medical University, No. 169, Dongshan street, Hushan Road, Jiangning District, Nanjing, 211100 China

**Keywords:** Lingguizhugan decoction (LGZG), Hepatic steatosis, STING, Inflammation

## Abstract

**Background:**

Stimulator of IFN genes (STING) is highly expressed in the livers of non-alcoholic fatty liver disease (NAFLD) patients and high fat diet (HFD) induced NAFLD mice model. The STING signaling-mediated inflammation has been shown to play a critical role in metabolic disorders. Lingguizhugan decoction (LGZG), a Traditional Chinese herbal decoction, has been applied to treat metabolic disorders for many years. However, whether LGZG can alleviate the progression of NAFLD through inhibiting inflammation remains unclear. This study was to determine the role of STING-mediated inflammation in the HFD-induced hepatic-lipid deposition treated with LGZG.

**Methods:**

The anti-inflammatory and anti-steatotic effects of LGZG in vivo were detected by H&E staining, immunofluorescence and immuno-chemistry. Mice bone-marrow-derived macrophages (BMDMs) and primary liver macrophages were treated with STING-specific agonist (DMXAA), LGZG and its critical components respectively. The treated culture supernatant of BMDMs and primary liver macrophages from each group was co-cultured with palmitic acid-treated mouse primary hepatocytes or mouse liver cell line AML-12 respectively to detect whether the activation of STING-mediated pathway is involved in the anti-steatotic effect of LGZG. The hepatocyte lipid deposition in vivo and in vitro were detected by oil red staining. Mitochondrial DNA release of mouse liver extracts were detected by real time PCR. The expression of proteins and inflammatory cytokines related to STING-TBK1-NF-κB pathway was detected by western blotting and ELISA.

**Results:**

LGZG significantly ameliorated HFD induced hepatic steatosis, oxidative stress, hepatic mitochondrial damage and mitochondrial DNA release, which was correlated with reduction of the expression level of STING as well as the infiltration of STING-positive macrophages in the livers of HFD fed mice. The critical components of LGZG directly inhibited the activation of STING-TBK1-NF-κB pathway in liver macrophages induced by DMXAA, LPS, thereby reducing the release of IFNβ and TNFα. Co-incubating the culture supernatant of LGZG treated liver macrophages and PA-stimulated hepatocytes significantly inhibited the PA-induced lipid deposition.

**Conclusion:**

This study demonstrates that LGZG can ameliorate HFD-induced hepatic-lipid deposition through inhibiting STING-TBK1-NF-κB pathway in liver macrophages, which provides novel insight for elucidating the molecular mechanism of LGZG alleviating HFD induced hepatic steatosis.

## Background

Non-alcoholic fatty liver disease (NAFLD) refers to liver injury caused by metabolic stress closely related to insulin resistance (IR) and genetic susceptibility. The disease spectrum includes non-alcoholic hepatic steatosis, nonalcoholic steatohepatitis (NASH), liver cirrhosis and hepatocellular carcinoma (HCC) [1]. In recent years, the incidence of NAFLD has risen sharply. The prevalence rate in the general population is 10–16%, and the detection rate in obese patients is as high as 38% [2]. NAFLD is a clinicopathological syndrome dominated by hepatocyte steatosis caused by genetic-environmental-metabolic stress related factors, and its pathogenesis has not been fully elucidated. It is currently believed to be closely related to obesity, aging, abnormal intestinal flora, diabetes, especially insulin resistance (IR).

Recently, a large number of studies have shown that inflammation plays a key role in the process of NAFLD [3]. Inflammatory factors and inflammatory response run through all stages of NAFLD progression [4, 5]. Alleviating liver inflammation can significantly slow down the progression of NAFLD [6–8]. Transmembrane protein 173 (TMEM173), also known as Stimulator of IFN genes (STING), is highly expressed in macrophages and becomes one of the core molecules involved in macrophages' resistance to virus invasion [9]. The STING signaling has been shown to be a critical role in metabolic disorders, anti-tumor immunity, infectious and inflammatory diseases through the recognition of bacterial DNA or self DNA [10]. When abnormal double-stranded DNA (such as bacterial, viral DNA or DNA released by damaged mitochondria, mtDNA) is swallowed by macrophages into the cytoplasm, cyclic guanosine phosphate and adenosine phosphate are activated to form cyclic guanosine phosphate-adenosine phosphate (cGAMP) [11]. STING on the endoplasmic reticulum will be recognized and binded to cGAMP and then activated [11]. The activation of STING pathway simultaneously triggers TANK-binding kinase 1 (TBK1), which induces the phosphorylation of both interferon regulatory factor 3 (IRF3) [11] and NF-κB pathway [12], subsequently increasing the expression of type I interferon (IFN) and TNF-α [11]. STING-mediated signaling pathways play a key role in both liver macrophage-induced inflammation and IR-induced glucose as well as lipid metabolism disorders [13, 14].

Lingguizhugan decoction (LGZG) is an ancient Chinese herbal formula from a classic book of TCM titled Shanghan Zabing Lun. LGZG has been applied to treat metabolic diseases for many years and exhibited effects in alleviating obesity, dyslipidemia, hypertension and hepatic injury [15–18]. Previous clinical studies have shown that LGZG can significantly alleviate liver steatosis, reduce TG, ALT and other biochemical indicators in NAFLD patients [15–18]. More importantly, studies have shown that LGZG elicit significantly anti-inflammatory effects [19]. However, whether LGZG can reduce lipid deposition in NAFLD via inhibiting STING signal pathway remains unclear. Here we proved that LGZG could reduce the release of inflammatory factors via inhibiting STING-mediated signaling pathways in liver macrophages, thereby ameliorating HFD-induced hepatic-lipid deposition in mice. This study will provide new insights for the expounding of the molecular mechanism of LGZG treatment in alleviating NAFLD.

## Materials and methods

### Reagents and cell lines

The four crude herbs, Poria cocos (PC, FuLing, Cat. No. 20200102-1), Ramulus Cinnamomi (RC, GuiZhi, Cat. No. 20200201-1), Rhizoma Atractylodis Macrocephalae (RAM, BaiZhu, Cat. No. 2011010) and Radix Glycyrrhizae (RG, GanCao, Cat. No. 20191001) were purchased from the Affiliated Hospital of Integrated Traditional Chinese and Western Medicine and authenticated by Professor Fangshi Zhu. All the crude drugs were morphologically authenticated according to Chinese Pharmacopoeia (2020 Edition). Reference substances including Poriaic acid, Cinnamaldehyde, Atractylenolide II and Glycyrrhizinate were purchased from Topscience Co. Ltd (Shanghai, China). Lipopolysaccharide (LPS), STING specific inhibitor C176 and STING specific agonist DMXAA were purchased from MedChemExpress (MCE, NJ, USA). High fat diet (HFD) was purchased from New Brunswick (Cat. No. D12492, NJ, USA). Antibodies against STING, F4/80, TBK1, Phosphorylated TBK1 (p-TBK1), TNFα, 8-OHdG, 4-hydroxy-2,3-E-nonenal (4-HNE) and 3-Nitrotyrosine (3-NT) were purchased from CST (CST, GER). Enzyme-linked immunosorbent assay (ELISA) kits were purchased from R&D (R&D Systems, US). DNA extraction kit, First Strand cDNA Synthesis Kit and 2 × SYBR Green/ROX qPCR Mix kit were purchased from Vazyme Biotech (Nanjing, Jiangsu, China). TRIzol reagent was purchased from Invitrogen (Carlsbad, CA, USA). Microplate assay kits for Blood gamma glutamyl transferase (GGT), alanine aminotransferase (ALT), aspartate aminotransferase (AST), triglyceride (TG) and total cholesterol (TC) measurement were purchased from Beyotime Biotechnology (Beyotime, Beijing, China). ELISA Kits were purchased from R&D Systems. Alpha mouse liver 12 (AML-12) cell line was purchased from National Collection of Authenticated cell cultures (Shanghai, China).

### Preparation of LGZG

LGZG, consisting of PC, RC, RAM and RG in the ratio of 4:3:3:2, were boiled twice with 10 times of deionized water (ddH_2_O, w/v) for 1 h per time. The raw drug extracts were concentrated and lyophilized. The lyophilized powder was weighed and dissolved in ddH_2_O for further use. The HPLC analysis was performed to identify the main compounds in LGZG (5 mg/mL) as previously reported [20]. Briefly, HPLC analysis was performed to identify the main compounds in LGZG. Chromatographic separation was performed using an Waters XBridge C18 column (4.6 mm × 250 mm, 5 μm; Waters, USA) at 30 °C. The mobile phase consisted of water containing 0.1% Formic acid (A) and Methanol (B). The gradient program was set as follows: 0–5 min, 5–12% B; 5–20 min, 12–14% B; 20–40 min, 14–30% B; 40–50 min, 30–35% B; 50–60 min, 35–43% B; 60–70 min, 43–50% B; 70–75 min, 50–75% B; 75–90 min, 75–85% B; 90–95 min, 85–95% B; 95–95.1 min, 95–5% B. The mobile phase flow rate was 1 mL/min, and the sample injection volume was 100 μL.

### Animals, feeding, and treatment

6 to 8-week-old male C57BL/6J mice (18 ~ 20 g)were purchased from the Chinese Academy of Sciences, Shanghai Experimental Animal Center and feed in Experimental Animal Center, Affiliated Hospital of Integrated Traditional Chinese and Western Medicine (Nanjing, China). Mice were group-housed in a pathogen-free animal facility under a 24 h light/dark cycle with free access to water and food. The experiment protocols were reviewed and approved by the Animal Ethics Committee of Affiliated Hospital of Integrated Traditional Chinese and Western Medicine with reference to European Community guidelines for the use of experimental animals. The mice were fed with standard chow diet (10% kcal from fat, set as the normal control group, n = 9) or a high-fat diet (HFD, D12492 rodent diet) composed of 60% fat and 20% carbohydrate (n = 36). After 8 weeks feeding, the HFD fed mice were randomly divided into four groups (n = 9) including Model (Mod), LGZG-L (low dose of LGZG, at a dose of 11 g/kg/day), LGZG-H (high dose of LGZG, at a dose of 22 g/kg/day), and C176 (STING specific inhibitor, C-176, 6.7 mg/kg/day). Mice were fed with HFD and orally administered LGZG for an additional 9 weeks and then sacrificed for sampling. The mice body weight and fasting blood glucose (FBG) levels were measured weekly before sacrifice. Intra peritoneal glucose tolerance test (IPGTT) and insulin tolerance tests (ITTs) were performed at 3 days before sacrifice. For IPGTT, mice were fasted for 8 h and then intraperitoneally injected with glucose (2 mg/g body weight). The blood glucose levels were measured at 0, 15, 30, 60, 90, and 120 min after glucose challenge respectively. For the insulin tolerance test (ITT), mice were fasted for 6 h and then intraperitoneal injected with human insulin (0.75 U/kg body weight). The blood glucose level was measured at 0, 30, 45, 60, 90 and 120 min after insulin injection respectively. At the end of experiment, blood samples for further assays were collected through abdominal heart puncture. The freshly separated liver tissues were quickly divided into several aliquots and stored in a − 80 °C refrigerator and 4% paraformaldehyde respectively for further use. Blood GGT, ALT, AST, TG and TC were measured by commercially available assay kits according to the standard manual.

### Histology

Liver specimens were fixed in 4% paraformaldehyde, embedded in paraffin, and cut into 4 μm thick sections. Sections were stained with hematoxylin and eosin (H&E) according to a standard procedure for assessment histopathological changes. For Oil Red O staining, the fixed liver specimens were embedded in optimum cutting temperature compound (OCT), and cut into 10 μm thick sections. Sections were stained with 0.5% Oil Red O solution according to a standard procedure for assessment lipid accumulation. The degree of hepatic lipid deposition and oil red O positive area were quantified with Image J software.

### Immunofluorescence and immunohistochemistry

Paraffin-embedded mouse liver blocks from different groups were sliced into 4 μm-thick sections for immunofluorescence and immunohistochemistry staining. The antibodies against STING (1:200, CST, 16029T), F4/80 (1:200, CST, 70076S), 3-Nitrotyrosine (1: 100, Abcam, ab61392), 4 Hydroxynonenal (1: 100, Abcam, ab48506) and 8-OHdG (1:100, Abcam, ab48508) were used according to manufacturer's protocol. The fluorescence intensity was analyzed by using Image J software and normalized by nuclei fluorescence intensity (6-diamidino-2-phenylindole, DAPI, blue).

### Isolation of mtDNA from liver tissue extracts

Cytosolic mtDNA content was measured according to the previous work [21]. Briefly, freshly separated mice livers from different groups were divided into two aliquots. One aliquot was used to extract total DNA as the control for total mtDNA by using the DNA extraction kit according to the standard manual (Axygen US). The other one was used to extract cytosolic DNA. For the extraction of cytosolic DNA, the liver tissue was homogenized in 500 μL buffer (150 mM NaCl, 50 mM Hepes, 25 μg/mL digitonin, pH 7.4) and then incubated for 10 min to permeabilize the plasma membrane. The homogenate was centrifuged (980×*g*, 5 min) for 3 times to pellet intact cells. The cytosolic supernatants were further centrifuged at 17,000×*g* for 25 min to pellet any remaining cellular debris, yielding cytosolic preparations free of nuclear, mitochondrial, and endoplasmic reticulum contamination. DNA was then isolated from these pure cytosolic fractions by using the DNA extraction kit according to the standard manual.

### Real time PCR (qPCR)

For detection of mtDNA, qPCR was performed on both whole-cell extracts and cytosolic fractions using primers of nuclear DNA (Tert) and primers of mtDNA (Dloop1 to 3, mtND4, and Cytb, Table 1), and the cycle threshold (CT) values obtained for mtDNA abundance for whole-cell extracts served as normalization controls for the mtDNA values obtained from the cytosolic fractions [21]. The detailed protocols was described in Juli Bai et al.’s work (Juli Bai et al. PNAS, 2017) [21]. Data was analyzed using the ΔΔCT relative quantification method. Primers sequences and corresponding genes were shown in Table 1.

**Table 1:** Primer pair sequences

Gene	Accession no	Primer	Sequence5′-3′
β-Actin	NM_007393.5	Forward Reverse	GTTGGTTGGAGCAAACATC CTTATTTCATGGATACTTGGAATG
Tert	NC_000079.6	Forward Reverse	CTAGCTCATGTGTCAAGACCCTCTT GCCAGCACGTTTCTCTCGTT
mtDNA loop 1	NC_005089	Forward Reverse	AATCTACCATCCTCCGTGAAACC TCAGTTTAGCTACCCCCAAGTTTAA
mtDNA loop 2	NC_005089	Forward Reverse	CCCTTCCCCATTTGGTCT TGGTTTCACGGAGGATGG
mtDNA loop 3	NC_005089	Forward Reverse	TCCTCCGTGAAACCAACAA AGCGAGAAGAGGGGCATT

### Cell separation, culture and drug treatment

Bone marrow cells were isolated from free-fed C57 mice and differentiated into bone-marrow-derived macrophages (BMDMs) as previously described [22]. Briefly, bone marrow cells were isolated from the tibias and femurs of the mice and cultured in DMEM containing 10% fetal bovine serum and 20% L929 culture supernatant for 6–8 days to obtain the BMDMs. The mouse primary hepatocytes and liver macrophages were isolated according to Meital Charni-Natan et al.’s work (Meital Charni-Natan et al., Nature protocols, 2020) [23]. Briefly, the mouse liver tissue was perfused in situ and digested with 0.5 g/L collagenase. The homogenized liver was filtered through a 70 μm cell strainer and then centrifuged at 50*g* for 5 min at 4 °C to pellet the fraction of hepatocytes. The supernatant was harvested and centrifuged at 300*g* for 5 min, and the pellet was collected and resuspended in PBS. The suspension was then layered on top of a density cushion of 30/60 discontinuous Percoll (Sigma, US) and centrifuged at 900*g* for 15 min to obtain the primary liver macrophages. For the detection of STING activation, the primary liver macrophages and B MDM cells were treated with DMXAA (75 μg/mL) for 24 h. Details are refer to Luo et al.’s work [13]. After incubation, the cell lysates from different groups were harvest respectively for the detection of expression of STING and other related proteins. Furthermore, the cell culture supernatant from each group were collected for the detection of IFNβ and TNF by ELISA.

For hepatocytes and macrophage co-culture study, the BMDMs and the primary liver macrophages were seeded in 6 well cell culture plate (5 × 10^5^ cells per well) and pre-treated with (7.5% NaHCO3) or different doses LGZG (LGZG-H: 10^–3^ g/mL; LGZG-L: 10^–4^ g/mL), Poriaic acid (60 μM), Cinnamaldehyde(40 μM), Atractylenolide II (50 μM) and Glycyrrhizinate (250 μM) for 2 h respectively as previously reported [19]. The concentration of these compounds is based on their cytotoxicity detected by CCK8 assay (data not shown). Then the cells were incubated with DMXAA (75 μg/mL) for 24 h in the absence or presence of LPS (100 ng/mL, dissolved in 1 × phosphate buffered saline, PBS). The culture supernatant from each group were harvest and respectively added to PA-stimulated hepatocytes at a dilution ratio of 1:10 and then incubated for another 48 h and assayed for hepatocyte fat deposition and inflammatory responses as previously reported [13]. The lipid deposition was detected by Oil Red O staining.

### Immunoblotting

For the immunoblotting, mice liver homogenates from and cultured cells were lysed with RIPA buffer. The denatured samples were loaded for SDS-PAGE, transferred, incubated with antibodies and visualized with enhanced chemiluminescence. The antibodies against F4/80, STING, TBK1, pTBK1, TNFα and tubulin were used. Ratios of p-TBK1 to TBK1 were normalized to Tubulin.

### Statistical analysis

For cell studies, data are representative of three independent experiments. The Western blot images were semi quantified with the Image J program. Statistical analysis of the data was performed using GraphPad Prism 7. Significance was assessed by performing an unpaired two-tailed Student’s t test as indicated in individual figures. Quantitative data are presented as Mean ± SEM. Statistical significance was performed using one/two-way ANOVA followed by multiple-comparison test. Within each row, compare columns *p < 0.05, **p < 0.01, ***p < 0.001, ****p < 0.0001, ns = not statistically significant.

## Results

### Identification of chemical compounds in LGZG by HPLC

**Fig. 1:**
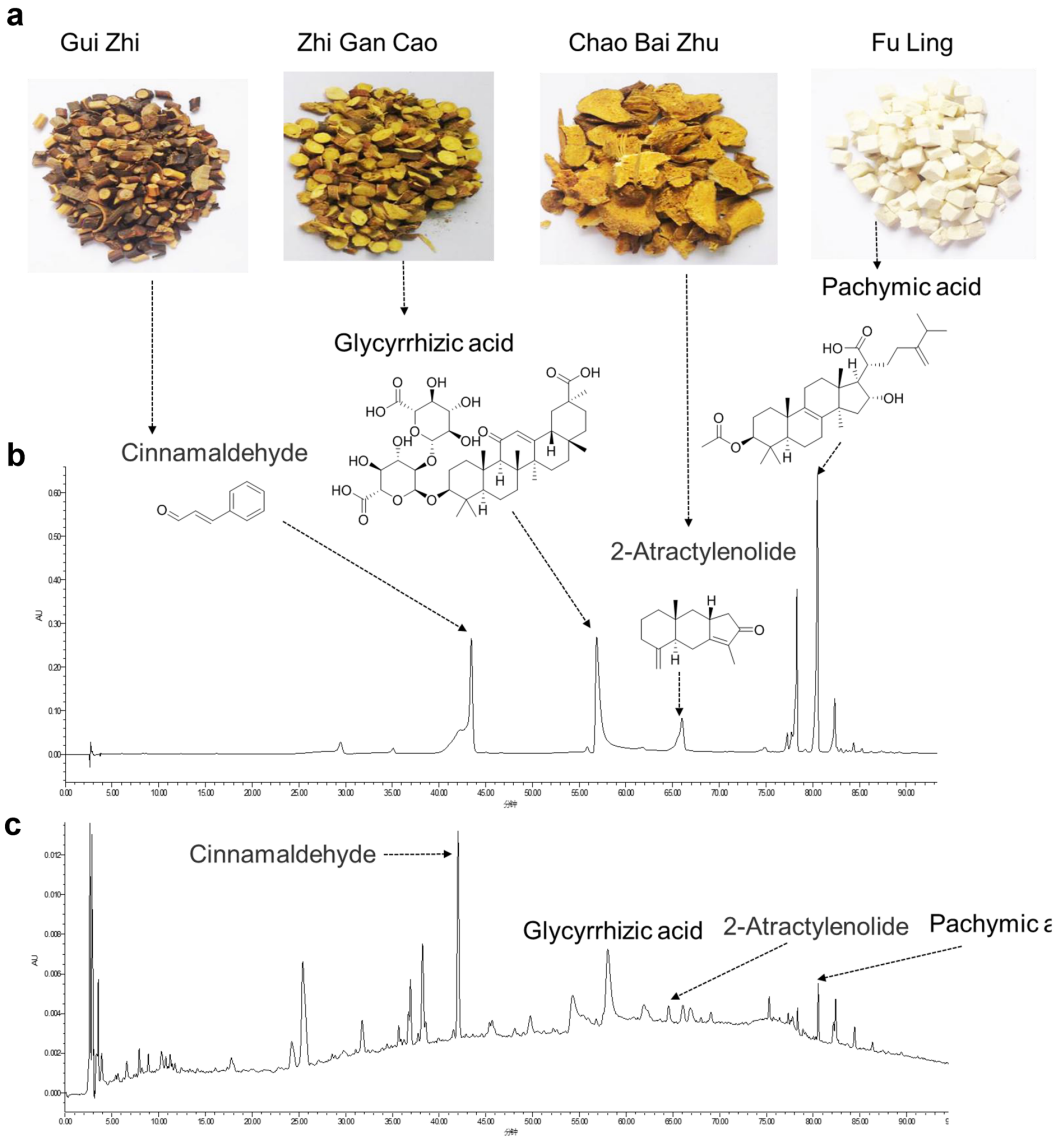
Identification of chemical compounds in LGZG by HPLC. **a** Images represent the four components that make up LGZG. **b** HPLC chromatograms of the mixture of Cinnamaldehyde, Glycyrrhizic acid, 2-Atractylenolide and Pachymic acid at 237 nm. **c** HPLC chromatograms of the LGZG at 237 nm

In the HPLC experiment, 4 compounds from FuLing, GuiZhi, BaiZhu and GanCao were detected in the LGZG (Fig. 1a). The 4 compounds were tentatively characterized based on their formula and retention times. According to the Chinese Pharmacopoeia, Cinnamaldehyde, Glycyrrhizinate, 2-Atractylenolide and Pachymic acid were used as references to verify the composition of LGZG. The representative HPLC chromatograms of standards and LGZG were shown in Fig. 1b, c. These results show that LGZG contains the characteristic peaks corresponding to 4 kinds of standard products. Surprisingly, among these 4 compounds, Cinnamaldehyde is the most abundant component in LGZG (Fig. 1c).

### LGZG ameliorated HFD induced hepatic lipid metabolic disorder in mice

**Fig. 2:**
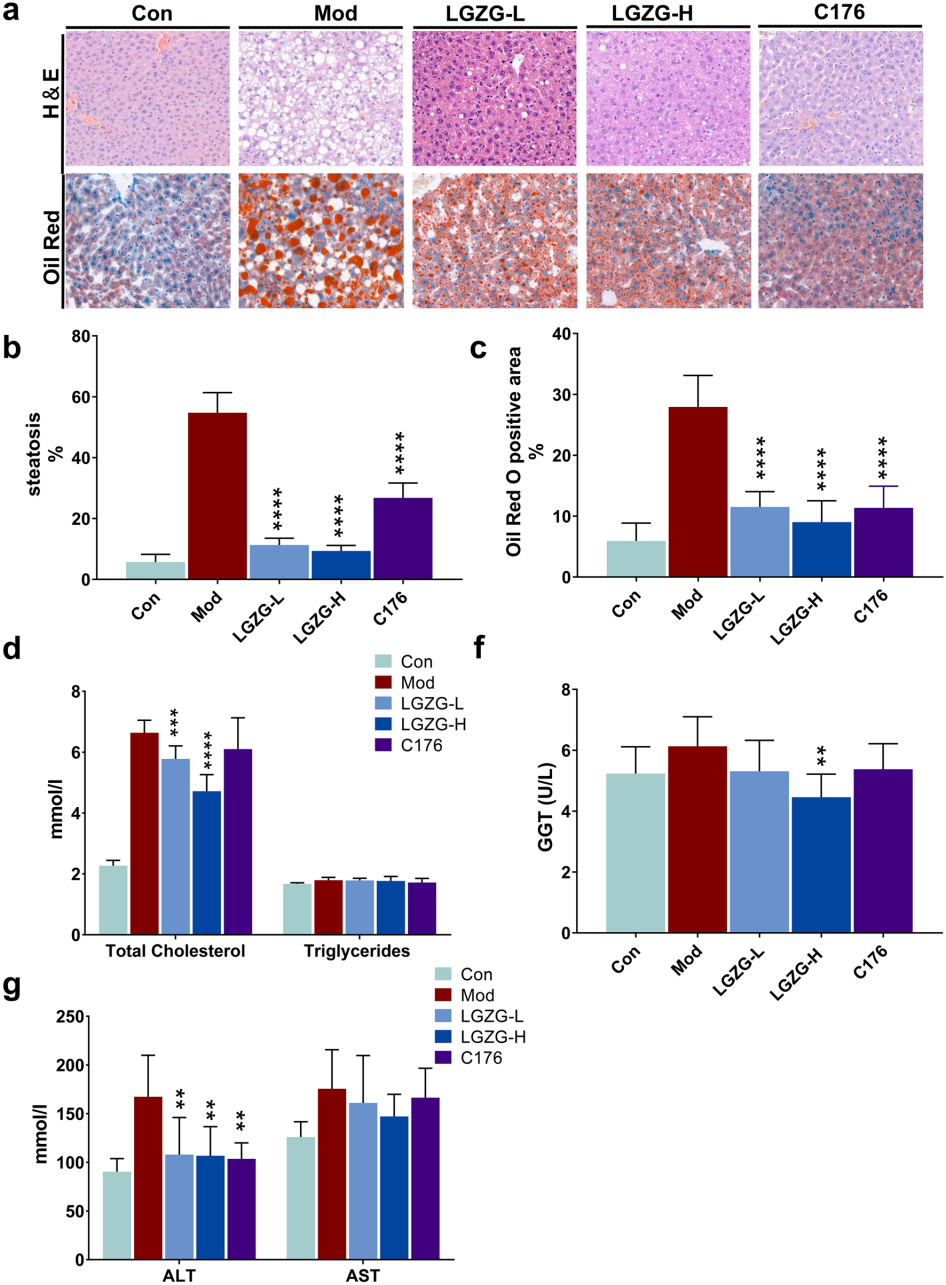
Effects of LGZG on HFD induced hepatic lipid metabolic disorder in mice. **a** Representative H&E-stained (up) and oil red stained images(down) of liver tissues from control and treated mice. Quantification of steatosis area (**b**) and oil red positive area (**c**). **d**, **e** The blood concentrations of total cholesterol (TC), triglyceride (TG, **e**), gamma glutamyl transferase (GGT) (**e**), alanine aminotransferase (ALT, **f**) and aspartate aminotransferase (AST, **f**). The data are expressed as mean ± SD (n = 9). *P < 0.05, **P < 0.01, ***P < 0.001, ****P < 0.001 vs. Mod group. The white scale bars represent 50 μm

To determine whether LGZG can alleviate HFD-induced hepatic lipid metabolic disorder, liver sections were subjected to pathological staining. The H&E and Oil-red O staining results showed that LGZG treatment significantly reduced HFD-induced hepatic-lipid deposition (p < 0.001, n = 7, Fig. 2a–c). The hepatic steatosis rate of the HFD mice model group was up to 50%, while after LGZG treatment, it reduced to about 10% (Fig. 2b, c). Interestingly, the STING specific inhibitor C176 can also significantly reduce the hepatic lipid deposition in HFD mice with a hepatic steatosis rate of about 27% (Fig. 2a–c). Furthermore, LGZG significantly decreased the blood TC level but did not affect TG in the HFD mice (Fig. 2d). In addition, LGZG can significantly reduce the blood GGT, ALT and AST to ease HFD-induced liver damage (p < 0.01, Fig. 2e, f). These results suggest that both LGZG and STING-specific blocker ameliorate HFD induced hepatic lipid metabolism disorder in mice. Statistical significance was performance using one-way ANOVA followed by multiple-comparison test. *p < 0.05, **p < 0.01, ***p < 0.001, ns = not statistically significant vs. model group.

### LGZG reduced HFD induced insulin resistance in mice

**Fig. 3:**
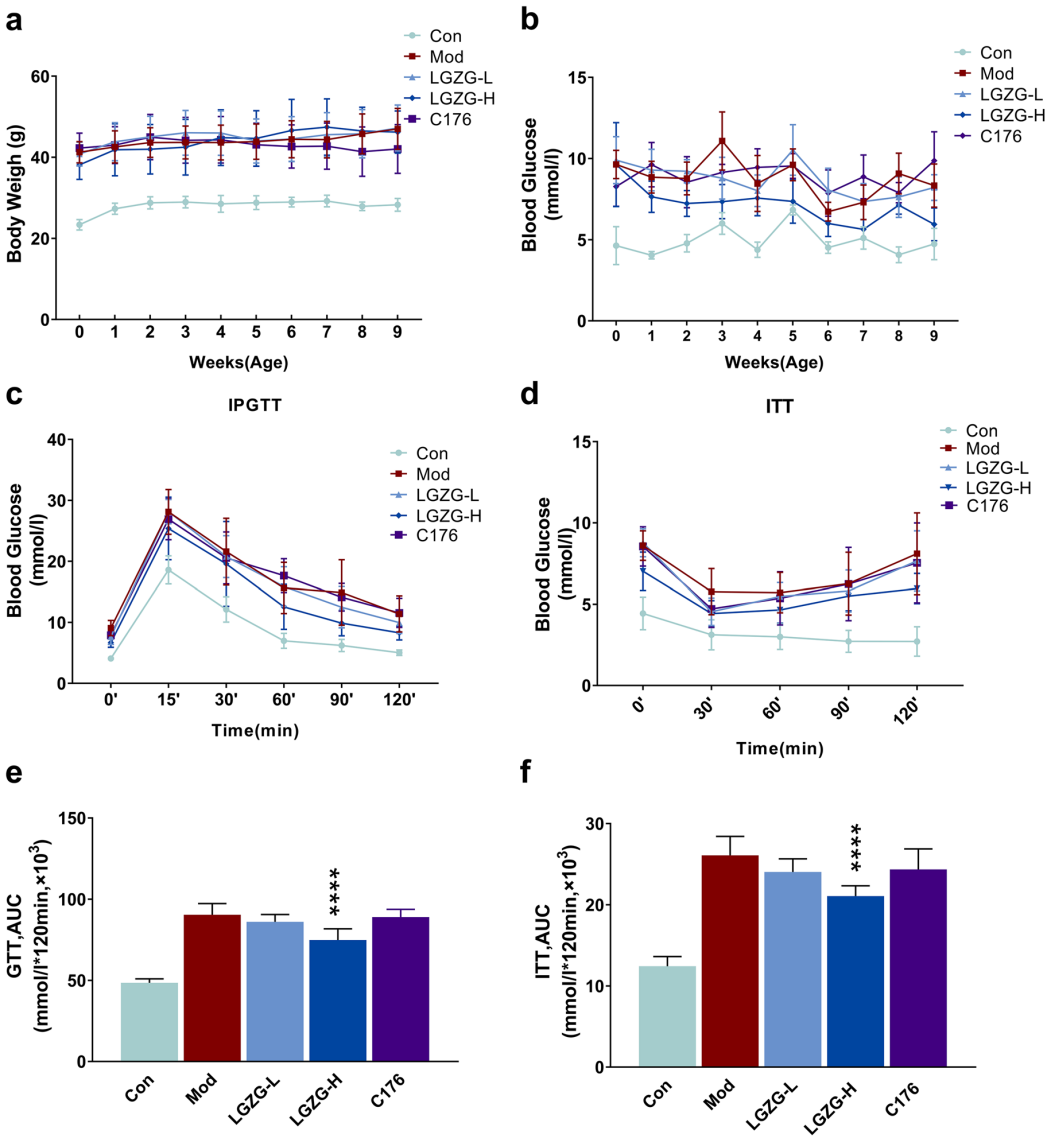
Effects of LGZG on hyperglycemia, glucose tolerance and insulin tolerance in HFD-fed mice. **a** Body weight changes during the LGZG treated period. Weight is measured once a week (n = 9 per group). **b** Changes in fasting blood glucose levels after a switch to HFD The blood glucose level is measured once a week. **c**, **d** Glucose tolerance (**c**) and insulin tolerance (**d**) after 9 weeks of LGZG treatment on HFD. For the IPGTT, mice were injected with glucose (2 mg/g body weight) after an 8-h fasting. AUC of the IPGTT (**e**) and ITT (**f**). For the ITT, mice were injected with insulin (0.75 U/kg body weight) after a 6-h fasting. The data are expressed as mean ± SD (n = 9). *P < 0.05, **P < 0.01, ***P < 0.001, ****P < 0.001 vs. Mod group

HFD induces obesity. We firstly tested the effect of LGZG on the body weight of HFD mice. The results revealed that LGZG had little effect on HFD induced body weight increasing (Fig. 3a). However, LGZG can significantly inhibit HFD-induced hyperglycemia in a dose dependent manner (Fig. 3b). Insulin resistance (IR) is closely related to liver inflammation and lipid metabolism [5]. To detect whether LGZG could reduce HFD induced IR, we further tested the effect of LGZG on glucose tolerance and IR in HFD mice by IPGTT and ITT assay. The IPGTT results showed that HFD induced significant glucose tolerance (48.46 ± 2.48 vs 90.44 ± 6.89, con vs. mod, Fig. 3b) and IR (12.42 ± 1.19 vs 26.09 ± 2.33, con vs. mod, Fig. 3c) in mice. The blood glucose levels in all groups peaked at 15 min after intra peritoneal glucose loading and then decreased. Compared with the model group, the blood glucose of HFD mice in the LGZG treatment group decreased rapidly after glucose loading, but the blood glucose of the model group (model) remained at a higher level (Fig. 3c). The ITT results revealed that after insulin injection, the blood glucose of HFD mice in the LGZG treatment group dropped rapidly in dose dependent manner, but the blood glucose of the model group had little change (Fig. 3d). The AUC of both GTT and ITT in the high dose of LGZG group was significantly lower than that in the model group (p < 0.001, Fig. 3e, f). However, STING-specific blocker had little effect on HFD induced glucose tolerance and IR in mice. These results suggest that LGZG ameliorate HFD induced glucose tolerance and IR in mice. Furthermore, HFD induced glucose tolerance and IR might not be STING dependent. Statistical significance was performed using one/two-way ANOVA followed by multiple-comparison test. *p < 0.05, **p < 0.01, ***p < 0.001, ns = not statistically significant vs. model group.

### LGZG alleviated hepatic mitochondrial damage and oxidative stress in HFD-fed mice

**Fig. 4:**
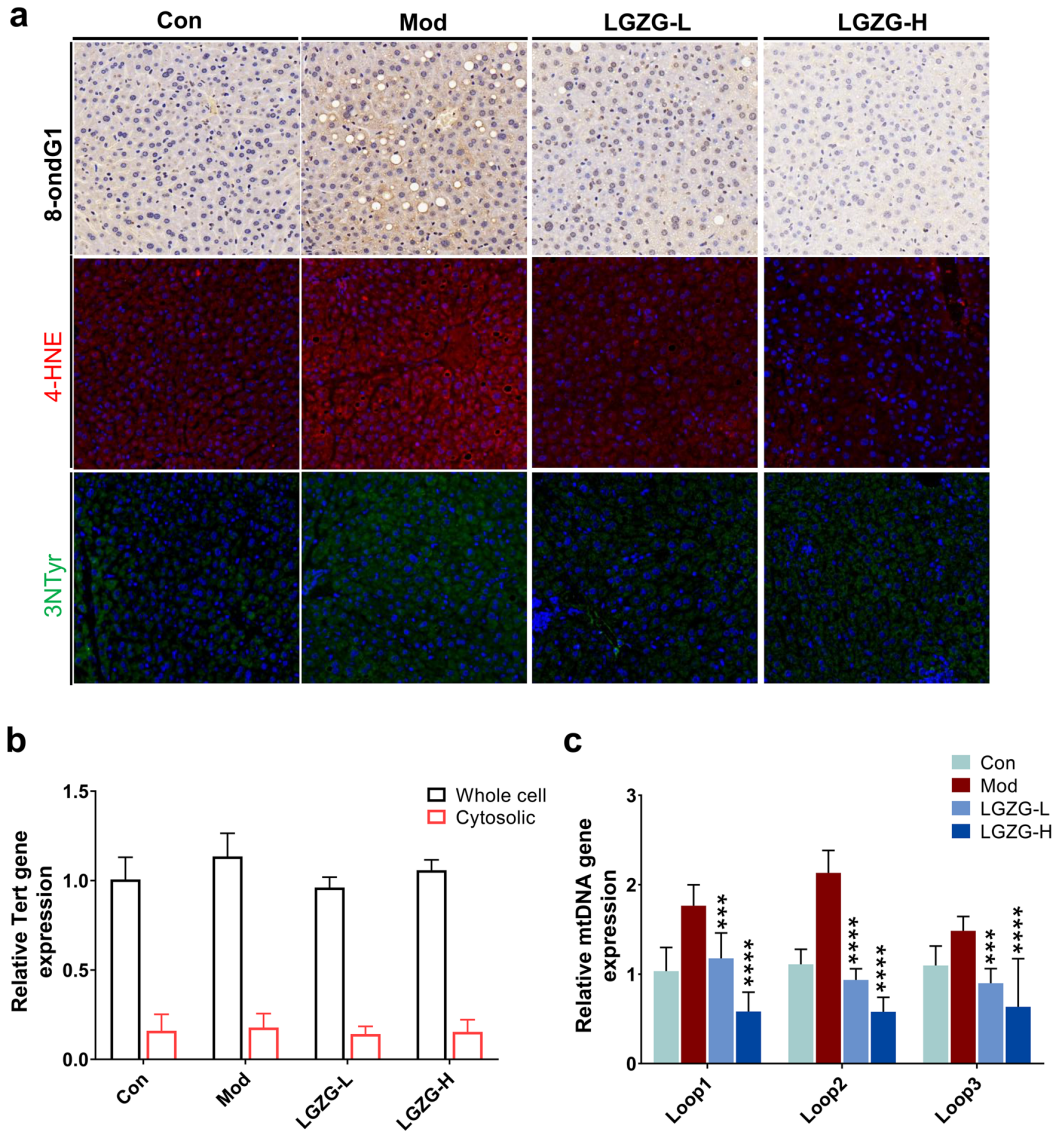
Effects of LGZG on liver mitochondrial damage and oxidative stress in HFD-fed mice. **a–c** Representative Immunohistochemistry and Immunofluorescence images of liver sections from control and treated mice. For the staining of the expression of oxidative stress related markers, Livers sections were immune-stained with antibodies against 8-OHdG (**a**, DAPI staining), 4HNE (**a**, red fluorescent) and 3-NT (**a**, green fluorescent). **b**, **c** The relative expression of Nuclear-encoded Tert gene (**b**) and Cytosolic mtDNA content (**c**) in mice livers from control and treated mice. The relative expression of these genes were detected by qPCR and calculated with 2^−△△CT^. The data are expressed as mean ± SD (n = 9). *P < 0.05, **P < 0.01, ***P < 0.001, ****P < 0.001 vs. Mod group. The white scale bars represent 50 μm

HFD induced obesity and IR can cause mitochondrial damage, which result in the release of oxidative free radicals as well as oxidative stress (OS). Increased OS in turn cause mitochondrial DNA (mtDNA) damage including mtDNA deletion. 8-hydroxy-2′-deoxyguanosine (8-OHdG), 4-Hydroxynonenal (4-HNE) and 3-nitrotyrosine (3-NT) are usually used as OS biomarkers in human liver diseases [24]. Oxidative damage occurs permanently in lipids of cell membranes, proteins and DNA whereas the 8-OHdG is one of the predominant forms of specific markers of DNA damage and RNA oxidation [24]. The 4-HNE is one of the most cytotoxic products of the lipid peroxidation cascade and exerts various effects on cell signaling cascades. The 3-NT, a marker of protein oxidative damage, is formed from peroxynitrite, a highly reactive free radical generated from nitric oxide (NO) and superoxide [25]. To detect the effect of LGZG on HFD induced OS, we compared the expression of 8-OHdG, 4-HNE and 3-NT in the liver extracts from different groups of HFD mice. The results of immunohistochemistry and immunofluorescence showed that HFD significantly increased the expression of 8-OHdG, 4-HNE and 3-NT whereas LGZG significantly reduced their expression in liver (Fig. 4a). OS further aggravates mitochondrial damage and the release of the mitochondrial DNA (mtDNA). To detect mtDNA copy number in the cytosol, we purified total DNA from the cytosolic fraction of hepatocyte freshly isolated from mice of different group. The purity of the cytosolic fraction was confirmed by cell-fractionation studies, which showed no contamination with the nuclear markers and Tert (Fig. 4b). The qPCR result revealed that mtDNA levels were significantly decreased in the cytosol of hepatocyte from LGZG treated groups compared with model group (p < 0.001, Fig. 4c). These results suggest that LGZG could significantly reduce HFD-induced mitochondrial damage and OS. Statistical significance was performed using one/two-way ANOVA followed by multiple-comparison test. *p < 0.05, **p < 0.01, ***p < 0.001, ns = not statistically significant vs. model group.

### LGZG reduced liver macrophage infiltration and STING expression in HFD-fed mice

**Fig. 5:**
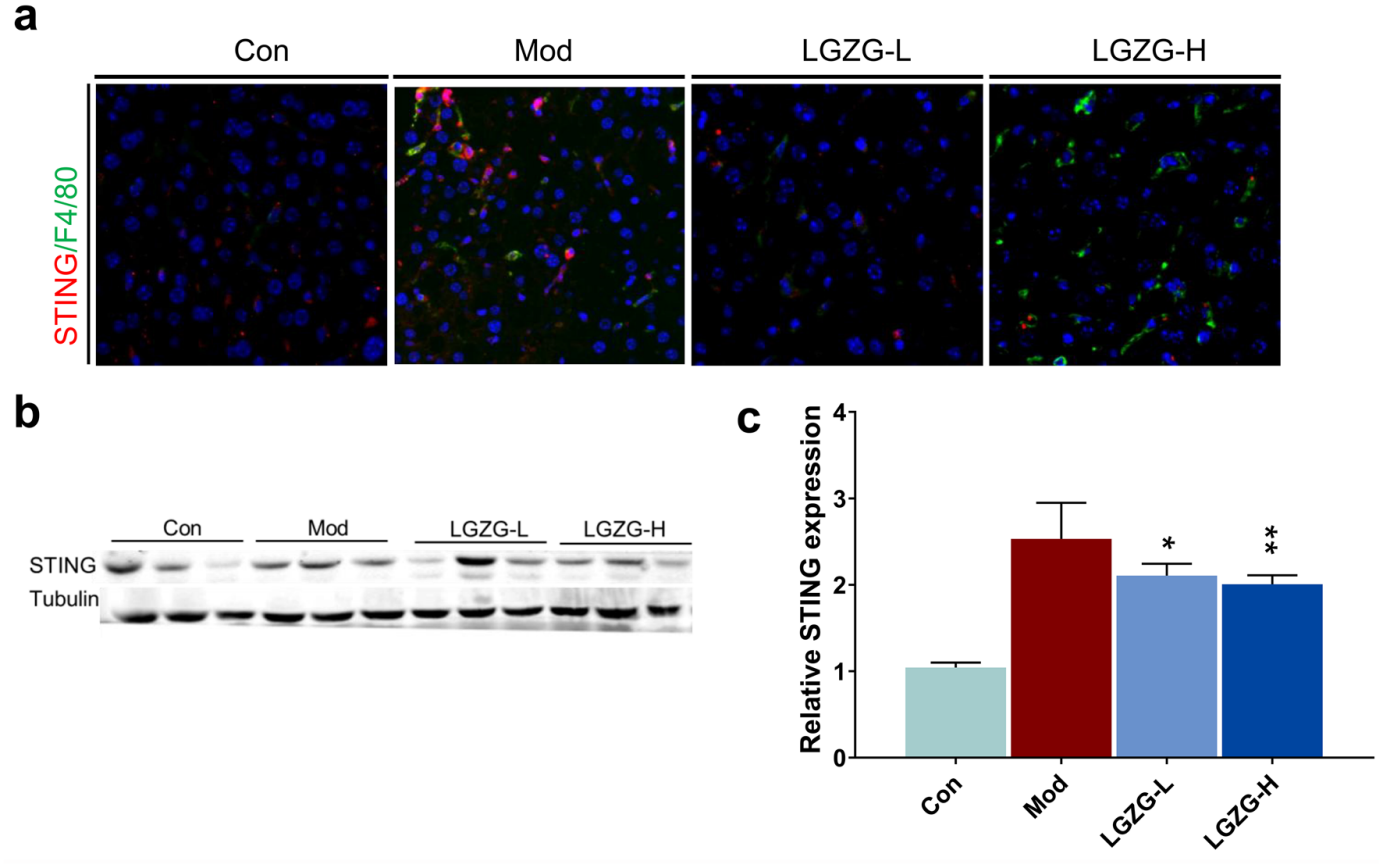
Effects of LGZG on liver macrophage infiltration and STING expression in HFD-fed mice. **a** Representative Immunofluorescence images of liver sections from control and treated mice. For the staining of STING and F4/80, Liver sections were double-immunostained with antibodies against STING and F4/80. The merged images including staining of STING (red), F4/80 (green), nuclei (blue) shown in **a**. **b**, **c** Immunoblotting analysis of the expression of STING in livers from control and treated mice. The relative expression were calculated based the Gray value of immunoblotting band (**c**). The data are expressed as mean ± SD (n = 3). *P < 0.05, **P < 0.01, ***P < 0.001, ****P < 0.001 vs. Mod group. The white scale bars represent 50 μm

HFD-induced mitochondrial damage and OS in the liver can lead to the release of mtDNA [14]. While the released mtDNA can be swallowed by hepatic-macrophages, which activates STING-mediated pathways and induces hepatic-inflammation [14, 26]. To investigate the effect of LGZG on the expression of STING in vivo, we test the expression of STING in liver from different groups of HFD fed mice by immunofluorescence and immunoblotting. Immunofluorescence results showed that compared with normal liver (control), liver macrophages (F4/80 positive cells, F4/80^+^, green) in model group increased significantly (Fig. 5a). Furthermore, STING mainly expressed in liver macrophages (STING positive and F4/80 positive cells, STING^+^ F4/80^+^ cells), and its expression was significantly increased in model group (Fig. 5a, b). However, after LGZG treatment, STING^+^ F4/80^+^ cells in the liver of HFD mice are significantly reduced (p < 0.01, Fig. 5b, c). These results suggest that LGZG reduces liver macrophage infiltration and STING expression in HFD-fed mice. Furthermore, these results are consistent with previous work that the hepatic macrophages are the key cellular sources of STING in the liver [26]. Statistical significance was performance using one-way ANOVA followed by multiple-comparison test. *p < 0.05, **p < 0.01, ***p < 0.001, ns = not statistically significant vs. model group.

### LGZG and its critical components inhibited the activation of STING-mediated signaling pathways in BMDMs and the primary liver macrophages

**Fig. 6:**
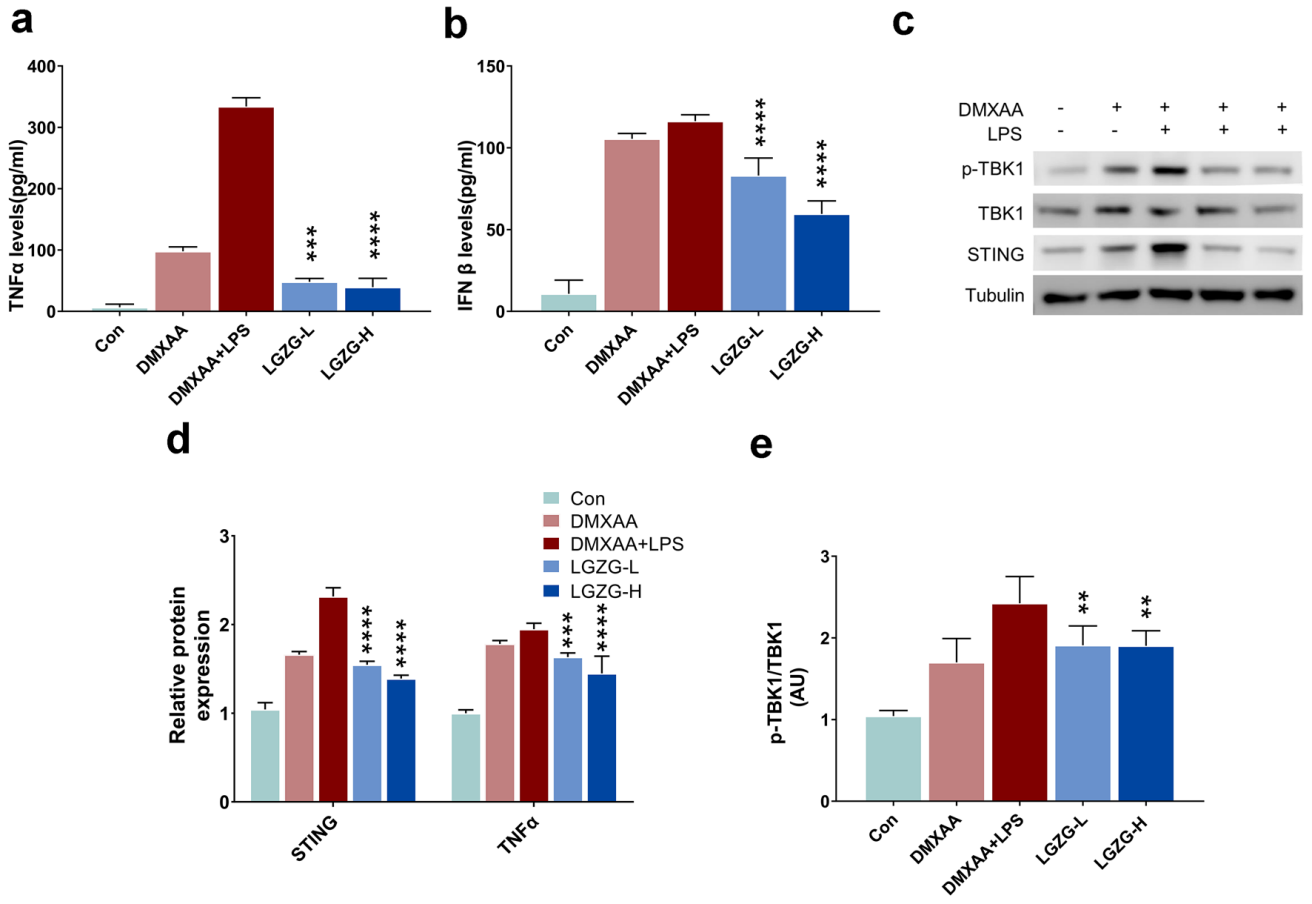
Effects of LGZG on STING-TBK1-NF-κB pathway in BMDMs. **a**, **b** Concentration of IFNβ (**a**) and TNFα (**b**) from the culture media of BMDMs treated with DMXAA, LPS and two doses of LGZG. BMDMs were treated with DMXAA (75 mg/mL) or control (7.5% NaHCO3) with or without LGZG (LGZG-L: 10^−4^or LGZG-H: 10^–3^ g/mL) for 24 h in the absence or presence of LPS (100 ng/mL). **c** Immunoblotting analysis of the expression of TBK1, p-TBK1 and STING. **d** Quantification of protein levels of STING and TNFα. **e** Quantification of p-TBK1/TBK1 levels. The data are expressed as mean ± SD (n = 3). *P < 0.05, **P < 0.01, ***P < 0.001, ****P < 0.0001 vs. DMXAA + LPS

**Fig. 7:**
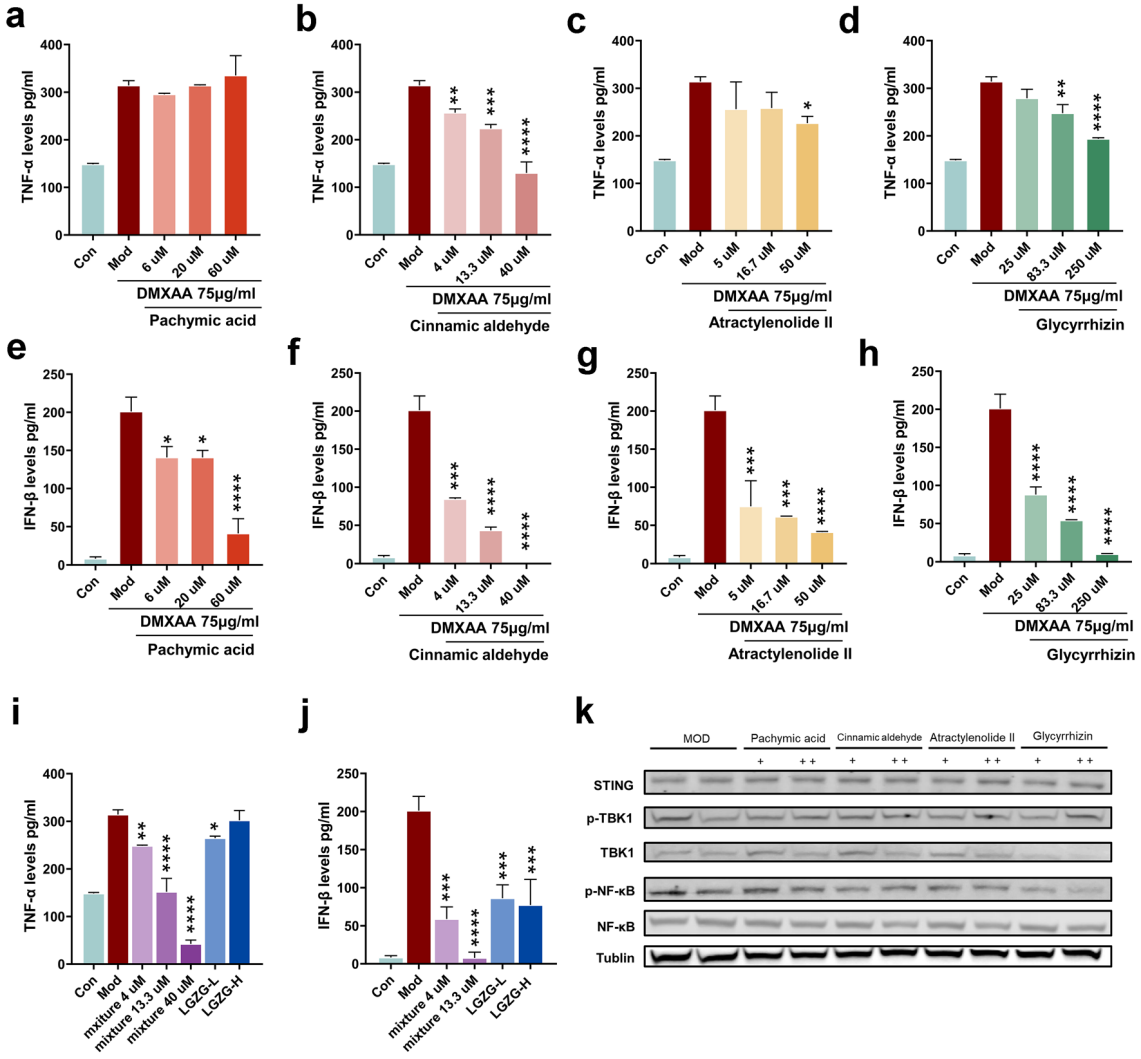
Identify the critical component of LGZG that inhibit the STING pathway in BMDMs. **a–d** Concentration of TNFα from the culture media of BMDMs treated with DMXAA and DMXAA co-cultured with different doses of Poriaic acid (**a**), Cinnamaldehyde (**b**), Atractylenolide II (**c**) and Glycyrrhizinate (**d**). **e–h** Concentration of IFNβ from the culture media of BMDMs treated with DMXAA and DMXAA co-cultured with different doses of Poriaic acid (**e**), Cinnamaldehyde (**f**), Atractylenolide II (**g**) and Glycyrrhizinate (**h**). **i**, **j** Concentration of TNFα and IFNβ from the culture media of BMDMs treated with DMXAA and DMXAA co-cultured with different doses of mixture of 4 compounds and LGZG. **k** Immunoblotting analysis of the expression of NF-κB, p NF-κB, TBK1, p-TBK1 and STING. The data are expressed as mean ± SD (n = 3). *P < 0.05, **P < 0.01, ***P < 0.001, ****P < 0.001 vs. DMXAA group

**Fig. 8:**
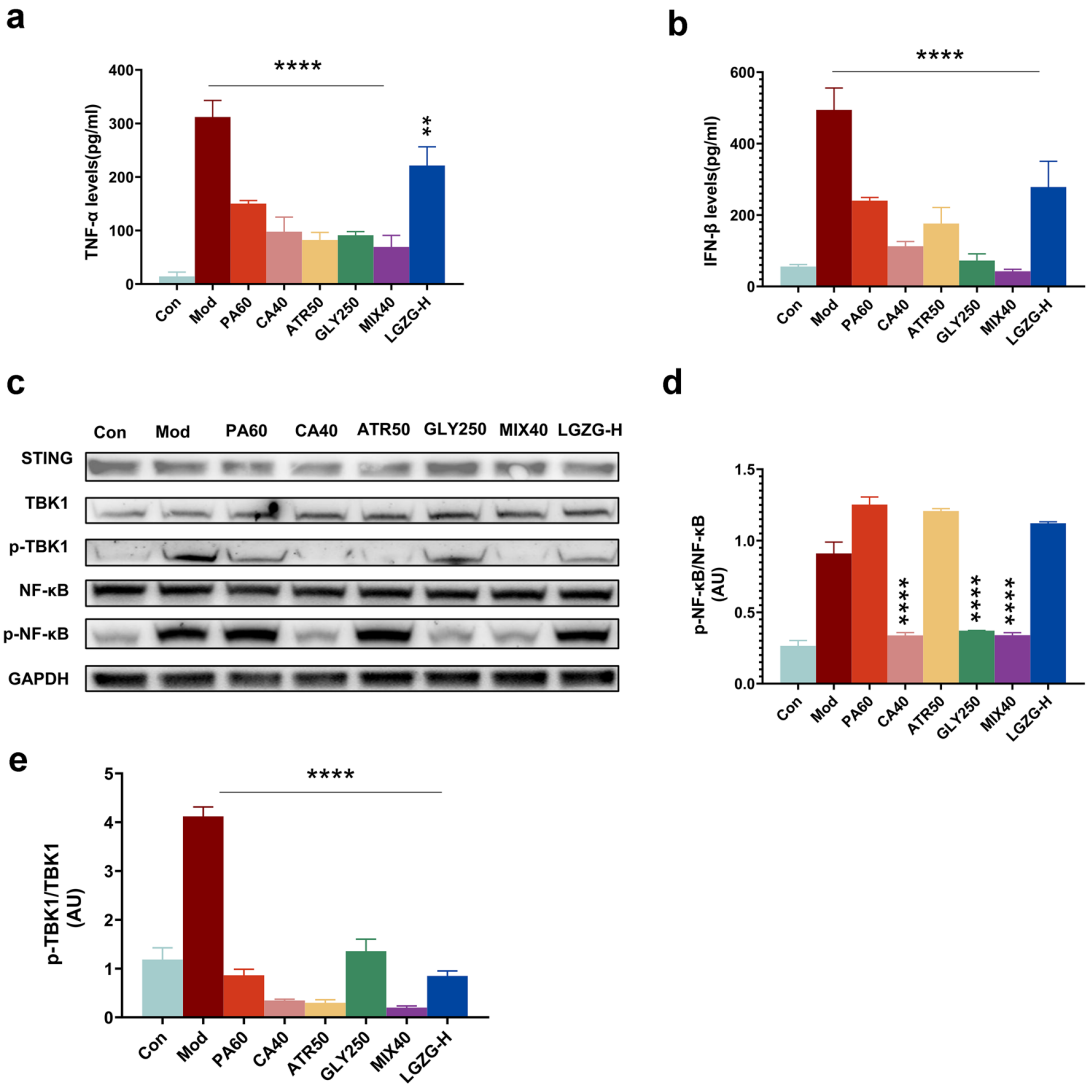
Identify the critical component of LGZG that inhibit the activation of STING pathway in liver macrophages. **a**, **b** Concentration of TNFα (**a**) and IFNβ (**b**) from the culture supernatant of liver macrophages treated with DMXAA and DMXAA co-cultured with 10^–3^ g/ml LGZG (LGZG-H), 60 μM Poriaic acid (PA60), 40 μM Cinnamaldehyde (CA40), 50 μM Atractylenolide II(ATR50), 250 μM Glycyrrhizinate(GLY250) and 40 μM mixture of 4 components of LGZG(MIX40). **c**, **d** Immunoblotting analysis of the expression of NF-κB, pNF-κB, TBK1, p-TBK1 and STING. Quantification of pNF-κB/NF-κB levels (**d**) p-TBK1/TBK1 levels (**e**). The data are expressed as mean ± SD (n = 3). *P < 0.05, **P < 0.01, ***P < 0.001, ****P < 0.001 vs. DMXAA group (Model)

Studies have shown that STING-mediated signaling pathways such as STING-TBK1-NF-κB pathway play important roles in regulating the pro-inflammatory response of macrophages [27, 28]. The STING specific agonist DMXAA significantly enhanced the LPS-induced inflammatory response in macrophages, which result in the increasing of the release of IFNβ and the phosphorylation of JNK p46 and NFκB p65 [29]. To detect whether LGZG can regulate STING-mediated signal pathway in macrophages and inhibit macrophages induced inflammation, we tested the effect of LGZG on the STING-TBK1-NF-κB signaling pathway in isolated mice BMDMs. The ELISA results showed that the DMXAA and LPS significantly increased the release of IFNβ and TNFα while LGZG dose dependently inhibited DMXAA and LPS induced release of these cytokines (p < 0.001, Fig. 6a, b). Furthermore, DMXAA and LPS up-regulated the expression of STING and the phosphorylation of TBK1 whereas LGZG dose dependently inhibited this upregulation in BMDMs (p < 0.001, Fig. 6c–e). These results indicate that LGZG can directly inhibit the activation of the STING-TBK1-NF-κB pathway in macrophages and reduce the release of inflammatory factors. To further identify the key components of LGZG that inhibit the STING mediate pathway, we tested the effect of the four reference compounds, including Poriaic acid, Cinnamaldehyde, Atractylenolide II and Glycyrrhizinate from LGZG on the STING-TBK1-NF-κB signaling pathway in isolated mice BMDMs. The ELISA results revealed that Cinnamaldehyde, Atractylenolide II and Glycyrrhizinate can dose-dependently inhibit the release of TNFα induced by DMXAA stimulation in mouse BMDMs (p < 0.01, Fig. 7a–d). Furthermore, Poriaic acid, Cinnamaldehyde, Atractylenolide II and Glycyrrhizinate all can inhibit the release of IFNβ (p < 0.01, Fig. 7e–h). Among these four compounds, Glycyrrhizic acid and Cinnamaldehyde elicit stronger inhibitory activity than the other two compounds in the release of inflammatory cytokines. Figure 7i, j compare the anti-inflammatory activity of a mixture of these 4 compounds in equal proportions and LGZG. The results revealed that both the mixture and LGZG can significantly inhibit the release of inflammatory cytokines in dose dependent manner with 13.3 μM mixture almost abolished the release of IFNβ (Fig. 7i, j). Surprisingly, the western blot results showed that all these 4 compounds cannot inhibit the expression of STING in BMDM. One reasonable explanation is that LGZG may contain other components which can inhibit the expression of STING in macrophages. However, Cinnamaldehyde and Glycyrrhizic acid significantly inhibit the expression of p-TBK1 and p-NF-κB in a dose-dependent manner (statics based on the p-TBK1/TBK1 and p-NF-κB/ NF-κB, p < 0.01, Fig. 7k). Since liver macrophages are composed of tissue resident macrophages (Kupffer cells) and infiltrating macrophage cells, we further test the effect of LGZG and its critical components on the primary liver macrophages. The results showed that LGZG and its critical components significantly inhibit the release of TNFα and IFNβ induced by DMXAA stimulation, which is consistent with the results observed in BMDMs (p < 0.01, Fig. 8a, b). The western blotting analysis showed that Cinnamaldehyde and Glycyrrhizic acid can also significantly reduced the expression of pTBK1 and p-NF-kB in liver macrophages (***p < 0.001, Fig. 8c–e). However, Atractylodes lactone (ATR), Poriaic acid (PA) and LGZG have little inhibitory effect on the expression of p-NF-kB (Fig. 8c, d). These results suggest that the critical components of LGZG can inhibit the activation of STING-mediated signaling pathways in both BMDMs and liver macrophages. Furthermore, due to the complex composition of Chinese herbal prescription, the unknown ingredients in LGZG might affect the inhibitory effect of the anti-inflammatory components of LGZG on the expression of p-NF-kB in vitro. Statistical significance was performed using one/two-way ANOVA followed by multiple-comparison test. All the groups compare with DMXAA group, *p < 0.05, **p < 0.01, ***p < 0.001, ns = not statistically significant.

### Macrophages treated with LGZG and its critical components reduced lipid deposition in PA-induced high-lipid hepatocyte model by inhibiting STING-mediated pathway

**Fig. 9:**
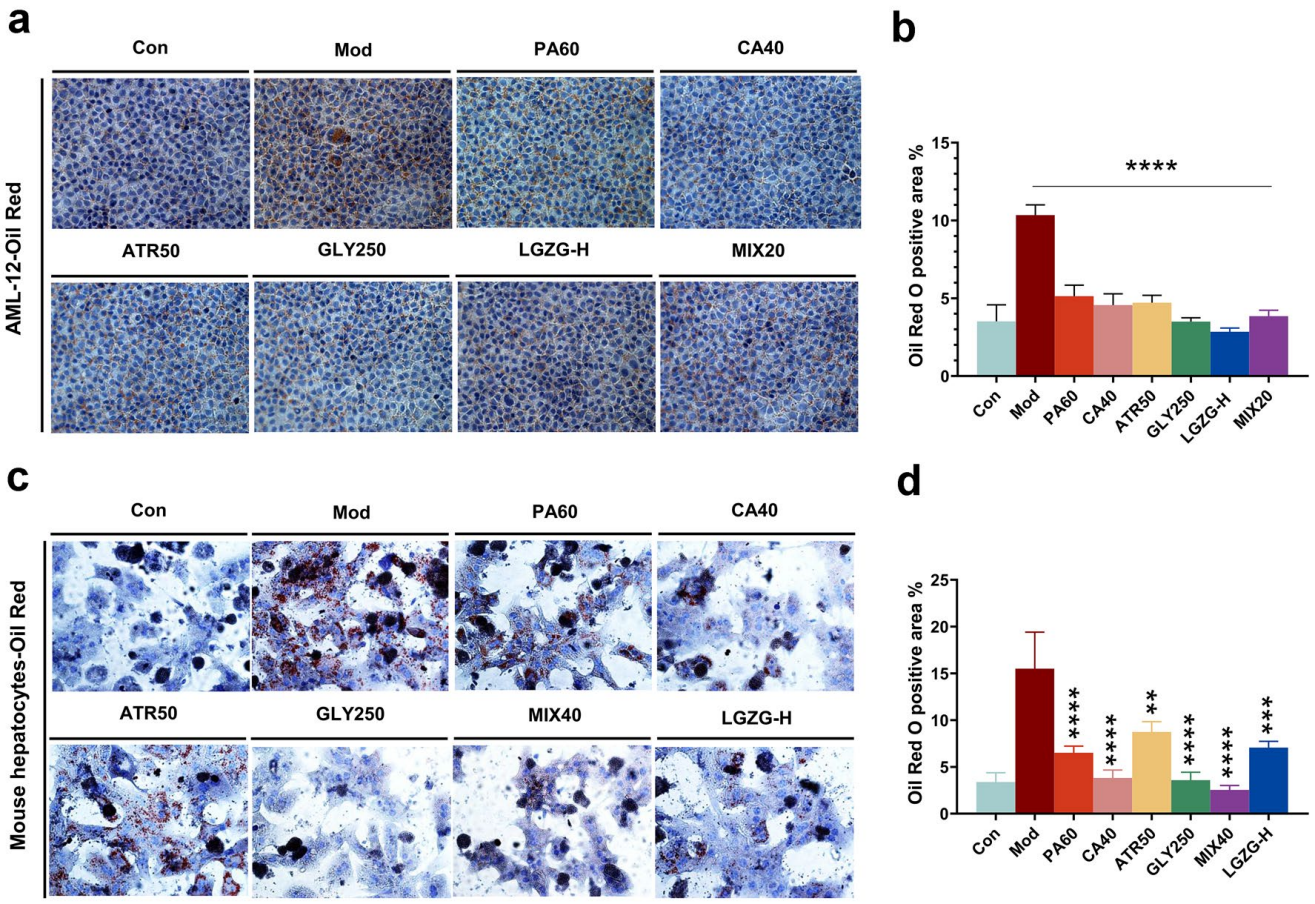
Culture supernatant of BMDMs/liver macrophages treated with LGZG Decoction reduced lipid deposition in liver cells. **a** Images of hepatocyte fat deposition of co-cultures of supernatant of treated BMDMs from different groups and AML-12 cells. **b** Bar graph displays quantification of fat content. **c** Images of hepatocyte fat deposition of co-cultures of supernatant of treated primary liver macrophages from different groups and mouse primary hepatocytes. **d** Bar graph displays quantification of fat content. ****P < 0.001 vs. Model (DMXAA) group

STING-driven macrophages have been reported to enhance hepatocyte fat deposition and proinflammatory responses [13, 26]. To recapitulate our in vivo findings and gain mechanistic insights, the culture supernatant from LGZG and its critical components treated or untreated BMDMs/liver macrophages were co-incubated with PA-stimulated AML-12 cells/mouse primary hepatocytes and assayed for hepatocyte fat deposition. The Oil Red O staining results revealed that the cell lipid deposition in AML-12 and mouse primary hepatocytes co-culture groups were significantly increased after the macrophages culture supernatant from DMXAA group (Model group) was added (Fig. 9a–d). However, after adding the culture supernatant from LGZG and its critical components (PA60: 60 μM Poriaic acid, CA40: 40 μM Cinnamaldehyde, ATR50: 50 μM Atractylenolide II, GLY250: 250 μM Glycyrrhizinate, MIX: 13.3 μM mixture of 4 compounds) treated BMDM to PA-stimulated hepatocytes, the cell lipid deposition significantly reduced (Fig. 9a–d). The effect of the LGZG and the mixture were better than that of any compound, which indicates that there may be a synergistic effect between the various components of the LGZG formula. These results suggest that STING activation enables macrophages to generate factors that can enhance hepatocyte lipid deposition while the critical components of LGZG can reduce cell lipid deposition induced by STING activation in macrophages.

## Discussion

Previous studies have shown that hepatic macrophages-mediated inflammation is one of the key factors leading to the progression of NAFLD and other metabolic disorders [5]. These provide possibilities for the promotion of the application of TCM containing anti-inflammatory component in the prevention and treatment of metabolic diseases. LGZG is widely used in the treatment of metabolic diseases such as NAFLD and Type 2 diabetes. Although LGZG has been reported to alleviate NAFLD via reducing OS, Beta-Oxidation and cholesterol secretion [18, 30], whether LGZG can alleviate NAFLD by inhibiting hepatic macrophage-mediated inflammation is not yet fully understood. Here we demonstrated that LGZG could significantly reduce the mitochondrial damage and OS in the liver of HFD fed mice. Furthermore, LGZG can inhibit inflammation mediated by STING activation in hepatic-macrophages, thereby alleviating HFD-induced liver lipid deposition.

STING, which closely related to inflammation, is highly expressed in hepatic macrophages (Kupffer cells) in both NAFLD patients and HFD-mice models [13]. The inflammatory response caused by activation of the STING-TBK1-NF-κB pathway in Kupffer cells promotes the progression of NAFLD [14]. mtDNA released by mitochondrial damage is one of the key ways to activate the STING- mediated pathway in hepatic macrophages [14]. The activation of STING-TBK1-NF-κB pathway can promote the release of a large number of inflammatory cytokines, including IFNβ and TNFα. Increased TNFα-producing hepatic macrophages are crucial for triggering NASH via monocyte recruitment [31]. IFNβ and TNFα released by hepatic macrophages not only aggravate mitochondrial damage and OS in hepatocytes, but also directly activate the JNK-NF-κB pathway in hepatocytes, thereby promoting lipid deposition [13]. These can explain why the inhibition of STING activation elicited by LGZG and STING specific inhibitor in hepatic-macrophages can reduce HFD induced liver lipid deposition.

LGZG contains a lot of anti-inflammatory components, such as tuckahoe polysaccharides, atractylodes polysaccharides which may down regulate the inducible nitric oxide synthase (iNOS) [32]. Our results suggest that the critical components of LGZG, such as Cinnamaldehyde and Glycyrrhizic acid can significantly inhibit the activation of STING pathway in macrophages. This may explain why LGZG could directly inhibit the LPS or STING specific agonist induced activation of STING-TBK1-NF-κB pathway in BMDMs. Furthermore, previous study showed that STING mediate neuro-inflammatory injury by suppressing AMPK pathway [33]. Therefore, another explanation is that some components in LGZG may activate the AMPK pathway, thereby inhibiting the inflammation induced by STING activation. Both of these two explanations are reasonable, which is consistent with the complex composition and multiple targets of LGZG.

We also found that LGZG reduced the HFD induced elevation of ALT and GGT while have little effect on AST. This is possibly because that GGT and ALT are two early stage indicators of HFD induced liver damage while AST is an advanced stage indicator of HFD induced liver damage. 8–12 weeks high-fat feeding usually develop an early stage NAFLD model. In our study, we started giving LGZG in 8 weeks, which may prevent the aggregation of HFD induced liver damage. Triglycerides are closely related to the composition of diet. Our results suggest that the high-fat diet used in this study may mainly affect the liver's cholesterol metabolism rather than Triglycerides. This can explain why triglycerides was not changed during the drug intervention.

Insulin resistance is another key factor for the progression of NAFLD, which is closely related to inflammation [5]. However, our results showed that LGZG can significantly improve HFD-induced glucose resistance and insulin resistance in mice, but the STING inhibitor C176 has little effect on insulin resistance and glucose resistance, which is consistent with previous work that diet-induced adipose insulin resistance and glucose intolerance is related to STING signaling but not dependent on it [34]. In contrast, the STING-mediated pathway in liver macrophages is more closely related to HFD-induced lipid deposition in hepatocytes.

## Conclusions

In summary, this study demonstrates that LGZG can alleviate HFD-induced hepatic steatosis through inhibiting the activation of STING in macrophages, which provides novel insight for elucidating the molecular mechanism of LGZG's anti-NAFLD effect.

## Data Availability

The data used to support the findings of this study are available from the corresponding author upon request.

## Abbreviations

LGZG: Lingguizhugan decoction
NAFLD: Non-alcoholic fatty liver disease
HFD: High fat diet
STING: Stimulator of IFN genes
BMDMs: Bone-marrow-derived macrophages
DMXAA: 5,6-Dimethylxanthenone-4-acetic acid
IR: Insulin resistance
LPS: Lipopolysaccharides
TBK1: TANK-binding kinase 1
IFNβ: Interferon beta
TNFα: Tumor necrosis factor alpha
NASH: Nonalcoholic steatohepatitis
HCC: Hepatocellular carcinoma
TCM: Traditional Chinese medicine
PPPM: Perspective of predictive, preventive and personalized medicine
TMEM173: Transmembrane protein 173
cGAMP: Cyclic guanosine phosphate-adenosine phosphate
IRF3: IFN regulatory factor 3
PC: Poria cocos
RC: Ramulus Cinnamomi
RAM: Rhizoma Atractylodis Macrocephalae
RG: Radix Glycyrrhizae
HPLC: High-performance liquid chromatography
FBG: Fasting blood glucose
IPGTT: Intra peritoneal glucose tolerance test
ITT: Insulin tolerance test
GGT: Gamma glutamyl transferase
ALT: Alanine aminotransferase
AST: Aspartate aminotransferase
TG: Triglyceride
TC: Total cholesterol
H&E: Hematoxylin and eosin
OCT: Optimum cutting temperature compound
3NT: 3-Nitrotyrosine
4HNE: 4 Hydroxynonenal
DAPI: 6-Diamidino-2-phenylindole
8-OHdG: 8-Hydroxy-2′-deoxyguanosine
PA: Palmitate
ROS: Reactive oxygen species

## References

[1] Huang TD, Behary J, Zekry A (2020). Non-alcoholic fatty liver disease: a review of epidemiology, risk factors, diagnosis and management. Intern Med J.

[2] Cotter TG, Rinella M (2020). Nonalcoholic fatty liver disease 2020: the state of the disease. Gastroenterology.

[3] Gehrke N, Schattenberg JM (2020). metabolic inflammation-a role for hepatic inflammatory pathways as drivers of comorbidities in nonalcoholic fatty liver disease?. Gastroenterology.

[4] Matulewicz N, Karczewska-Kupczewska M (2016). Insulin resistance and chronic inflammation. Postepy Hig Med Dosw.

[5] Shimobayashi M, Albert V, Woelnerhanssen B, Frei IC, Weissenberger D, Meyer-Gerspach AC (2018). Insulin resistance causes inflammation in adipose tissue. J Clin Investig.

[6] He L, Liu X, Wang L, Yang Z (2016). Thiazolidinediones for nonalcoholic steatohepatitis: a meta-analysis of randomized clinical trials. Medicine..

[7] Chu JW, Abbasi F, Lamendola C, McLaughlin T, Reaven GM, Tsao PS (2005). Effect of rosiglitazone treatment on circulating vascular and inflammatory markers in insulin-resistant subjects. Diab Vasc Dis Res.

[8] Lan W, Wang Z, Liu J, Liu H (2020). Methionyl-methionine exerts anti-inflammatory effects through the JAK2-STAT5-NF-κB and MAPK signaling pathways in bovine mammary epithelial cells. J Agric Food Chem.

[9] Barber GN (2015). STING: infection, inflammation and cancer. Nat Rev Immunol.

[10] Zhang X, Wu J, Liu Q, Li X, Li S, Chen J (2020). mtDNA-STING pathway promotes necroptosis-dependent enterocyte injury in intestinal ischemia reperfusion. Cell Death Dis.

[11] Hu HQ, Qiao JT, Liu FQ, Wang JB, Sha S, He Q (2020). The STING-IRF3 pathway is involved in lipotoxic injury of pancreatic β cells in type 2 diabetes. Mol Cell Endocrinol..

[12] Bai J, Liu F (2019). The cGAS-cGAMP-STING pathway: a molecular link between immunity and metabolism. Diabetes.

[13] Luo X, Li H, Ma L, Zhou J, Guo X, Woo S-L (2018). Expression of STING is increased in liver tissues from patients with NAFLD and promotes macrophage-mediated hepatic inflammation and fibrosis in mice. Gastroenterology.

[14] Yu Y, Liu Y, An W, Song J, Zhang Y, Zhao X (2019). STING-mediated inflammation in Kupffer cells contributes to progression of nonalcoholic steatohepatitis. J Clin Investig.

[15] Xu J, Wang R, You S, Zhang L, Zheng P, Ji G (2020). Traditional Chinese medicine Lingguizhugan decoction treating non-alcoholic fatty liver disease with spleen-yang deficiency pattern: Study protocol for a multicenter randomized controlled trial. Trials.

[16] Zhu M, Hao S, Liu T, Yang L, Zheng P, Zhang L (2017). Lingguizhugan decoction improves non-alcoholic fatty liver disease by altering insulin resistance and lipid metabolism related genes: a whole trancriptome study by RNA-Seq. Oncotarget.

[17] Yang L, Lin W, Nugent CA, Hao S, Song H, Liu T (2017). Lingguizhugan decoction protects against high-fat-diet-induced nonalcoholic fatty liver disease by alleviating oxidative stress and activating cholesterol secretion. Int J Genomics.

[18] Liu T, Yang LL, Zou L, Li DF, Wen HZ, Zheng PY (2013). Chinese medicine formula Lingguizhugan decoction improves beta-oxidation and metabolism of fatty acid in high-fat-diet-induced rat model of fatty liver disease. Evid Based Complement Altern Med..

[19] Xi F, Sang F, Zhou C, Ling Y (2012). Protective effects of Lingguizhugan decoction on amyloid-beta peptide (25–35)-induced cell injury: anti-inflammatory effects. Neural Regen Res.

[20] Hua D, Yang J, Meng Q, Ling Y, Wei Q, Wang Z (2021). Soufeng sanjie formula alleviates collagen-induced arthritis in mice by inhibiting Th17 cell differentiation. Chin Med.

[21] Bai J, Cervantes C, Liu J, He S, Zhou H, Zhang B (2017). DsbA-L prevents obesity-induced inflammation and insulin resistance by suppressing the mtDNA release-activated cGAS-cGAMP-STING pathway. Proc Natl Acad Sci USA.

[22] Xu H, Li H, Woo SL, Kim SM, Shende VR, Neuendorff N (2014). Myeloid cell-specific disruption of Period1 and Period2 exacerbates diet-induced inflammation and insulin resistance. J Biol Chem.

[23] Charni-Natan M, Goldstein I (2020). Protocol for primary mouse hepatocyte isolation. STAR protocols..

[24] Barbosa ML, de Meneses AM, de Aguiar RPS, de Castro e Sousa JM, de Carvalho Melo Cavalcante AA, Maluf SW (2020). Oxidative stress, antioxidant defense and depressive disorders: a systematic review of biochemical and molecular markers. Neurol Psychiatry Brain Res..

[25] Rose S, Melnyk S, Pavliv O, Bai S, Nick TG, Frye RE (2012). Evidence of oxidative damage and inflammation associated with low glutathione redox status in the autism brain. Transl Psychiatry..

[26] Maher JJ (2018). Macrophages steal STING from the infectious disease playbook to promote nonalcoholic fatty liver disease. Gastroenterology.

[27] Wang X, Rao H, Zhao J, Wee A, Li X, Fei R (2020). STING expression in monocyte-derived macrophages is associated with the progression of liver inflammation and fibrosis in patients with nonalcoholic fatty liver disease. Lab Invest.

[28] Ahn J, Barber GN (2019). STING signaling and host defense against microbial infection. Exp Mol Med.

[29] Downey CM, Aghaei M, Schwendener RA, Jirik FR (2014). DMXAA causes tumor site-specific vascular disruption in murine non-small cell lung cancer, and like the endogenous non-canonical cyclic dinucleotide STING agonist, 2'3'-cGAMP, induces M2 macrophage repolarization. PloS ONE..

[30] Sun J-p, Shi L, Wang F, Qin J, Ke B. Modified Linggui Zhugan Decoction (加味苓桂术甘汤) Ameliorates Glycolipid Metabolism and Inflammation via PI3K-Akt/mTOR-S6K1/AMPK-PGC-1 α Signaling Pathways in Obese Type 2 Diabetic Rats. Chinese Journal of Integrative Medicine. 2020. Epub ahead of print. 10.1007/s11655-020-3285-2 33211278

[31] Tosello-Trampont AC, Landes SG, Nguyen V, Novobrantseva TI, Hahn YS (2012). Kuppfer cells trigger nonalcoholic steatohepatitis development in diet-induced mouse model through tumor necrosis factor-α production. J Biol Chem.

[32] Guo L, Ma R, Sun H, Raza A, Tang J, Li Z (2018). Anti-inflammatory activities and related mechanism of polysaccharides isolated from Sargentodoxa cuneata. Chem Biodivers..

[33] Peng Y, Zhuang J, Ying G, Zeng H, Zhou H, Cao Y (2020). Stimulator of IFN genes mediates neuroinflammatory injury by suppressing AMPK signal in experimental subarachnoid hemorrhage. J Neuroinflamm.

[34] Mao Y, Luo W, Zhang L, Wu W, Yuan L, Xu H (2017). STING-IRF3 triggers endothelial inflammation in response to free fatty acid-induced mitochondrial damage in diet-induced obesity. Arterioscler Thromb Vasc Biol.

